# Utility of the Serum Cystatin C Level for Diagnosis of Osteoporosis among Middle-Aged and Elderly People

**DOI:** 10.1155/2019/5046852

**Published:** 2019-01-16

**Authors:** Satoshi Tanaka, Kei Ando, Kazuyoshi Kobayashi, Tetsuro Hida, Kenyu Ito, Mikito Tsushima, Masayoshi Morozumi, Masaaki Machino, Kyotaro Ota, Koji Suzuki, Taisuke Seki, Naoki Ishiguro, Yukiharu Hasegawa, Shiro Imagama

**Affiliations:** ^1^Department of Orthopaedic Surgery, Nagoya University Graduate School of Medicine, Nagoya, Aichi, Japan; ^2^Department of Orthopaedic Surgery, Japanese Red Cross Nagoya Daini Hospital, Nagoya, Aichi, Japan; ^3^Faculty of Medical Technology, School of Health Science, Fujita Health University, Aichi, Japan; ^4^Department of Rehabilitation, Kansai University of Welfare Science, Osaka, Japan

## Abstract

**Purpose:**

Osteoporosis is a common age-related disorder leading to increased bone fragility and risk of fracture. Early diagnosis of osteoporosis is a vital step in providing early therapeutic intervention. Serum cystatin C is a marker of early renal dysfunction, a predictor of cardiovascular and inflammatory diseases, and an inhibitor of the differentiation of osteoclast precursor cells. The purpose of this study was to evaluate the relationship between serum cystatin C and osteoporosis.

**Methods:**

We enrolled 46 subjects who attended a health checkup and underwent measurement of bone status by quantitative ultrasound and determination of the level of serum cystatin C. A comparative study was conducted between those with and without osteoporosis for all subjects collectively and in two subgroups aged <65 and ≥65 years.

**Results:**

Serum cystatin C levels were strongly correlated with age, creatinine, and bone status data, with significant negative correlations with stiffness, T-score, and percentage of young adult mean. Among patients with osteoporosis, serum cystatin C was significantly higher even after adjustment for age and sex, whereas no significant difference was noted in creatinine. For patients aged ≥ 65 years, serum cystatin C was significantly higher in subjects with osteoporosis, although there was no significant difference in age between normal subjects and those with osteoporosis.

**Conclusions:**

To the best of our knowledge, this is the first study to demonstrate an association between serum cystatin C and osteoporosis. Serum cystatin C is significantly higher in osteoporosis and in particular may be a useful marker for osteoporosis among middle and elderly people aged ≥ 65 years. Measurement of serum cystatin C can be carried out easily and may contribute to early diagnosis and treatment of osteoporosis.

## 1. Introduction

The World Health Organization (WHO) defines osteoporosis as a disease characterized by low bone mass and the microarchitectural deterioration of bone tissue, leading to increased bone fragility and risk of fracture [1, 2]. The incidence of osteoporosis has steadily increased in recent decades as a consequence of societal ageing, with approximately 200 million osteoporotic patients worldwide and approximately 8.9 million osteoporotic fractures [3]. These types of fractures, along with spinal kyphosis, are the most important factors underlying the reduced quality of life and survival of elderly patients [4, 5].

Osteoporosis is a common age-related disorder that often coexists with cardiovascular disease (CVD) and diabetes [6]. Consequently, the early diagnosis of osteoporosis is a vital step in providing early therapeutic intervention. Serum cystatin C is a sensitive indicator of early renal dysfunction and a strong independent predictor of CVD, diabetes-related mortality, and all-cause mortality [7, 8]. Recent evidence from the Japanese Orthopedic Association also suggested that serum cystatin C can be an early predictor of locomotive syndrome risk [9]. However, the association between serum cystatin C and osteoporosis remains unclear. Therefore, the aim of this study was to investigate the relationships between serum cystatin C and other factors related to osteoporosis in community-dwelling adults. Clarification of these relationships may be useful in the early diagnosis and treatment of osteoporosis.

## 2. Methods

### 2.1. Participants

The subjects were healthy Japanese volunteers who attended a basic health checkup in 2016 supported by the local Government. Since 1982, this checkup has been held annually in the town of Yakumo in a rural area of southern Hokkaido in Japan and consists of voluntary orthopedic and physical function examinations as well as internal medical examinations and psychological tests [10–12]. The inclusion criteria were (1) bone status data measured by quantitative ultrasound (QUS) bone densitometry at the calcaneus and (2) serum cystatin C level measured by a blood test. The exclusion criteria were as follows: a history of steroid use; severe osteoarthritis; history of fracture of the hip and spine; and treatment of osteoporosis, diabetes, kidney disease, and heart disease.

Among 555 participants who underwent a health checkup in 2016, 367 received a bone status examination by QUS. Of these, measurement of the serum cystatin C level was carried out in 54 participants who gave their written informed consent for sample collection, of whom 8 were subsequently excluded due to the above criteria. Therefore, 46 subjects were included in the final study. The study protocol was approved by the University Committee on Ethics in Human Research and by the Institutional Review Board of Nagoya University Graduate School of Medicine. All participants provided written informed consent and the study protocol was approved by the Institutional Review Board of Nagoya University Graduate School of Medicine. The study procedures were carried out in accordance with the principles of the Declaration of Helsinki.

### 2.2. Measurement of Bone Status Data

A water-bath ultrasound system (model A-1000 Plus II; Lunar, Madison, WI, USA) was used to measure bone status data at the calcaneus region of the independent foot [13, 14]. Stiffness (automatically calculated from broadband ultrasound attenuation and speed of sound), T-score, % young adult mean (YAM), Z-score, and % age-matched were recorded using a standard protocol supplied by the manufacturer.

### 2.3. Blood Test and Measurement of Serum Cystatin C Level

We obtained venous blood samples and performed a blood test. In this study, creatinine, which is one of the kidney function markers, and C-reactive protein (CRP), which is one of the markers of inflammation, were used for analysis. Serum cystatin C level was measured using a latex agglutination turbidimetric immunoassay (LA) method (LSI Medience Corporation, Tokyo, Japan). Biochemical analyses of the blood samples were performed using an autoanalyzer (JCA-RX20; Nihon Denshi, Tokyo, Japan).

### 2.4. Osteoporosis

Based on the WHO classification [1], healthy bone was defined as a T-score > −2.5 (normal group) and osteoporosis as a T-score ≤ –2.5 (osteoporosis group). Data were compared between these 2 groups. To consider the influence of age, this comparison was also performed in subgroups aged <65 and ≥65 years.

### 2.5. Statistical Analysis

Continuous variables are expressed as means [standard deviations (SDs)], and categorical variables are expressed as percentages. Correlations between serum cystatin C level and other variables were analyzed using Spearman's rank correlation coefficients. To investigate the relationship between serum cystatin C level and osteoporosis, the variables were compared between normal patients and those with osteoporosis using the Mann–Whitney* U* test, Fisher's exact test, and generalized linear model (GLM). The GLM analysis was adjusted for age and sex, which are known to be related to osteoporosis [1]. The threshold value for the predictive value of serum cystatin C level for the presence of osteoporosis was determined using the receiver operating characteristics (ROC) analysis. All statistical analyses were performed using SPSS Statistics v.25.0 software for Mac (IBM Corp., Armonk, NY, USA). A p value <0.05 was considered significant in all analyses.

## 3. Results

The mean age of the 46 subjects was 62.0 years (range, 40-88 years; 24 males and 22 females), mean body mass index (BMI) was 23.6 kg/m^2^, mean percent body fat (PBF) was 28.2%, and mean serum cystatin C was 0.77 mg/L. Demographic, blood test, and bone status data are shown in Table 1. BMI, PBF, creatinine, stiffness, Z-score, and % age-matched were found to be significant differences between males and females, and age, serum cystatin C, and prevalence of osteoporosis were not significantly different. Correlations between serum cystatin C and other variables are shown in Table 2. Serum cystatin C showed a strong significant positive correlation with age (r = 0.712, p < 0.001), creatinine (r = 0.612, p < 0.001), significant negative correlations with stiffness (r = –0.374, p = 0.010), T-score (r = –0.445, p = 0.002), and % YAM (r = –0.459, p = 0.001).

Serum cystatin C (p = 0.042) showed significant differences between normal and osteoporosis subjects (Table 3), although there was no significant difference in terms of age (p = 0.070), sex (p = 0.062), creatinine (p = 0.42), and CRP (p = 0.69). Furthermore, after controlling for age and sex using GLM analysis, serum cystatin C (p = 0.014, Table 3) was significantly higher in osteoporosis, with no significant difference between creatinine (p = 0.48) and CRP (p = 0.17). Subgroup analysis of patients aged <65 years revealed no significant difference in all variables, including serum cystatin C (p = 0.87, Table 4) between normal and osteoporosis subjects. However, at age ≥65 years, only serum cystatin C was significantly higher in osteoporosis and p = 0.043 (Table 4).

**Figure 1:**
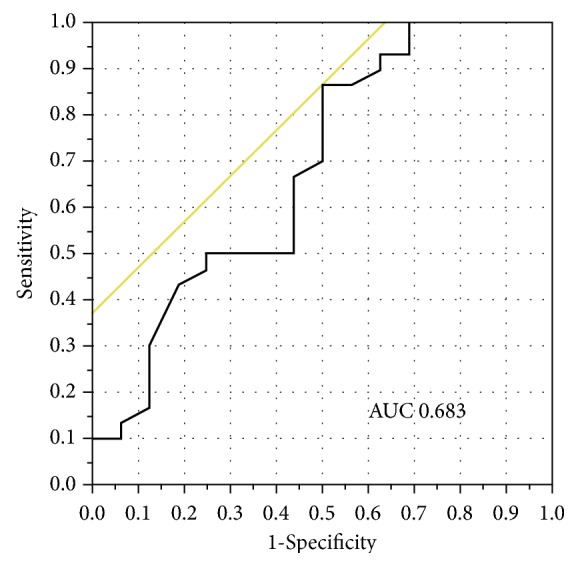
The receiver operating characteristic (ROC) curve for serum cystatin C level and osteoporosis. The area under the ROC curve was 0.683.

The ROC curve for the predictive value of the serum cystatin C level for the presence or absence of osteoporosis had an area under the curve of 0.683 (Figure 1, Table 5). From Youden's index [15], the cutoff value of serum cystatin C level was determined to be 0.840 mg/L for osteoporosis. A serum cystatin C level of ≥0.840 mg/L indicated the presence of osteoporosis with 50.0% sensitivity and 86.7% specificity (Table 5).

## 4. Discussion

There have been many previous reports involving biomarkers and predictors for osteoporosis [4, 15–19]. Bone resorption biomarkers, bone formation biomarkers, and regulators of bone turnover have all been implicated in previous research, and procollagen type 1 N-terminal propeptide has been identified as a sensitive and stable bone biomarker for the early detection of osteoporosis [4]. Osteoporosis develops as a complication of lifestyle-related diseases, with chronic obstructive pulmonary disease (COPD) being a risk factor among inflammatory diseases [20]. The association between COPD and serum cystatin C has been demonstrated previously [21], while serum cystatin C is a known risk factor for femoral neck fracture in elderly women [22]. However, no prospective study to date has investigated the relationship between serum cystatin C and osteoporosis in community-dwelling adults undergoing routine health checkup examinations.

Human cystatin C is a small protein composed of 122 amino acids that belongs to the cystatin family of papain-like cysteine protease inhibitors. It is broadly distributed and found in most bodily fluids [23]. Cystatin C primarily functions as a protease inhibitor and is a target of proteolytic degradation by cathepsin D and elastase [24]. Serum cystatin C level correlates with glomerular filtration rate [23], which is an important marker of kidney health and determinant of the progression of both diabetes and chronic kidney disease [25, 26]. Given its biological importance, serum cystatin C has also been linked to a number of other diseases [27].

To the best of our knowledge, this study is the first to investigate the relationship between serum cystatin C and osteoporosis. In this study, serum cystatin C was significantly higher in subjects with osteoporosis compared with normal subjects. Generally, it is known that osteoporosis tends to occur in older individuals and is more likely to occur in women than in men [1, 28]. The results of this study showed that age and sex were not significantly different between normal and osteoporosis subjects. However, as reported so far, osteoporosis tended to be older and was more likely to be females. Therefore, a comparative study was conducted by adjusting for age and sex using GLM. After adjustment, serum cystatin C was significantly higher in osteoporosis than normal subjects. However, creatinine which is one of kidney function markers and CRP which is one of inflammatory markers did not show any significant difference. Based on these results, serum cystatin C can be considered to be related to osteoporosis, without any effect from age and sex. Furthermore, subjects were divided into subgroups aged <65 and ≥65 years. Serum cystatin C did not differ significantly between normal and osteoporosis subjects aged <65 years, but was significantly higher in osteoporosis aged ≥65 years. Both creatinine and CRP did not show any significant difference in either subgroup. These results show that serum cystatin C is significantly associated with the presence of osteoporosis and, in particular, may be a novel predictor for osteoporosis in patients aged ≥65 years.

Previous studies have suggested a relationship between serum cystatin C and osteoclasts in osteoporosis, and it has been demonstrated that cystatin C reduces osteoclast formation by directly targeting osteoclast progenitor cells through an intracellular mechanism involving RANK signaling [29]. This suggests two possibilities: firstly, that osteoclast differentiation is increased in osteoporosis, and, in such conditions, cystatin C is not taken up by osteoclast progenitor cells, thus resulting in elevated serum cystatin C levels, and secondly that, in osteoporosis, the suppression of osteoclast differentiation may occur, thus resulting in a consequential increase in the expression of cystatin C. These mechanisms could explain why serum cystatin C increases in osteoporosis.

There are several limitations in this study which need to be considered. First, the number of subjects was relatively small. Second, we used QUS to measure bone status rather than dual X-ray absorptiometry (DXA). However, T-scores measured by QUS have been shown to correlate strongly with data derived from DXA [30], and measurement by QUS, which does not involve radiation exposure, is very useful for routine health checkups. Third, this study targeted residents in rural areas and there is a possibility of bias due to the inherent differences in living and working environments in rural areas when compared with urban areas. However, the results of this study are valuable as they are the first to demonstrate an association between serum cystatin C and osteoporosis.

## 5. Conclusions

In recent years, serum cystatin C has been recognized as an early renal function marker, and opportunities to measure it are increasing. As a result of this study, serum cystatin C is significantly correlated with osteoporosis and may be a particularly useful marker of osteoporosis among middle and elderly people aged ≥ 65 years. If the serum cystatin C value is high in people aged ≥ 65 years who have not been treated for osteoporosis, there is a possibility of osteoporosis. Therefore, the merit of serum cystatin C measurement is that it can lead to bone mineral density measurement at an early stage. Measurement of serum cystatin C is a simple procedure and would potentially allow us to prevent osteoporosis-related diseases.

## Figures and Tables

**Table 1:** Demographic characteristics, blood test, and bone status data for participants.

Variables	Total	Male	Female	p-value
Number of participants	46	24	22	
Age (years)	62.0 (14.3)	63.8 (14.5)	60.1 (14.3)	0.40
BMI (kg/m^2^)	23.6 (3.5)	24.2 (2.8)	22.5 (4.1)	***0.042*** **∗**
PBF (%)	28.2(7.1)	24.3 (5.2)	32.2 (6.7)	***< 0.001*** **∗** **∗**
Serum cystatin C (mg/L)	0.77 (0.19)	0.76 (0.18)	0.78 (0.21)	0.96
Creatinine (mg/dL)	0.75 (0.18)	0.84 (0.16)	0.66 (0.15)	***< 0.001*** **∗** **∗**
CRP (mg/dL)	0.09 (0.14)	0.13 (0.18)	0.06 (0.06)	0.066
Stiffness	80.1 (16.7)	88.3 (14.0)	71.1 (14.9)	***< 0.001*** **∗** **∗**
T-score	–1.68 (1.36)	–1.33 (1.2)	–2.05 (1.5)	0.054
% YAM	81.3 (15.1)	84.7 (13.4)	77.7 (16.3)	0.092
Z-score	0.18 (1.26)	0.65 (1.1)	–0.34 (1.3)	***0.008*** **∗** **∗**
% age-matched	102.9 (17.5)	109.6 (16.3)	95.6 (16.1)	***0.007*** **∗** **∗**
Prevalence of osteoporosis	34.8%	20.8%	50.0%	0.062

Italicized values indicate the significant difference.

*∗*p < 0.05, *∗∗*p < 0.01, Mann–Whitney *U* test, and Fisher's exact test.

Parameter values are shown as mean (standard deviation) or numbers.

BMI, body mass index; PBF, percent body fat; YAM, young adult mean.

**Table 2:** Correlation coefficients between variables and serum cystatin C levels.

Variables	Correlation coefficient (R)	p-value
Age (years)	0.712	***< 0.001*** **∗** **∗**
Sex (female)	−0.002	0.99
BMI (kg/m^2^)	0.054	0.72
PBF (%)	−0.066	0.67
Stiffness	−0.374	***0.010*** **∗**
T-score	−0.445	***0.002*** **∗** **∗**
% YAM	−0.459	***0.001*** **∗** **∗**
Z-score	−0.163	0.28
% age-matched	−0.153	0.31
Creatinine (mg/dL)	0.612	***< 0.001*** **∗** **∗**
CRP (mg/dL)	0.046	0.76

Italicized values indicate the significant difference.

*∗*p < 0.05, *∗∗*p < 0.01, and Spearman rank correlation coefficient analysis.

BMI, body mass index; PBF, percent body fat; YAM, young adult mean.

**Table 3:** Comparison between normal and osteoporosis patients.

	Non-adjusted			Age and sex-adjusted		
Variables	Normal	Osteoporosis	p-value	Normal	Osteoporosis	p-value
Number of participants	30	16		30	16	
Age (years)	59.1 (14.7)	67.4 (12.4)	0.070			
Sex (male/female)	19/11	5/11	0.062			
BMI (kg/m^2^)	23.8 (3.4)	22.7 (3.7)	0.69	24.0 (0.5)	23.2 (0.7)	0.33
PBF (%)	27.5 (7.3)	29.4 (6.8)	0.21	28.0 (0.9)	29.9 (1.2)	0.23
Serum cystatin C (mg/L)	0.72 (0.14)	0.87 (0.25)	***0.042*** **∗**	0.73 (0.03)	0.87 (0.04)	***0.014*** **∗**
Creatinine (mg/dL)	0.74 (0.17)	0.79 (0.21)	0.42	0.75 (0.01)	0.77 (0.02)	0.48
CRP (mg/dL)	0.08 (0.08)	0.14 (0.23)	0.69	0.09 (0.01)	0.12 (0.02)	0.17

Italicized values indicate the significant difference.

*∗*p < 0.05, Mann–Whitney *U* test, and Fisher's exact test.

Parameter values are shown as the mean (standard deviation) or numbers for nonadjusted data and corrected mean (standard error) or numbers of the mean for age adjusted data using generalized linear model.

BMI, body mass index; PBF, percent body fat.

**Table 4:** Comparison between normal and osteoporosis patients in subgroup analysis.

	Total			Aged < 65 years			Aged ≥ 65 years		
Variables	Normal	Osteoporosis	p-value	Normal	Osteoporosis	p-value	Normal	Osteoporosis	p-value
Number of participants	30	16		18	6		12	10	
Age (years)	59.1 (14.7)	67.4 (12.4)	0.070	48.7 (6.8)	55.3 (9.6)	0.10	74.6 (7.2)	74.7 (6.9)	0.97
Sex (male/female)	19/11	5/11	0.062	10/8	1/5	0.17	8/4	5/5	0.67
BMI (kg/m^2^)	23.8 (3.4)	22.7 (3.7)	0.32	23.9 (4.1)	22.2 (4.3)	0.25	23.5 (2.2)	23.1 (3.5)	0.77
PBF (%)	27.5 (7.3)	29.4 (6.8)	0.21	28.7 (8.0)	28.9 (6.5)	0.63	25.7 (6.0)	29.5 (7.0)	0.18
Serum cystatin C (mg/L)	0.72 (0.14)	0.87 (0.25)	***0.042*** **∗**	0.67 (0.10)	0.69 (0.13)	0.87	0.79 (0.15)	0.98 (0.24)	***0.043*** **∗**
Creatinine (mg/dL)	0.74 (0.17)	0.79 (0.21)	0.42	0.73 (0.16)	0.62 (0.15)	0.18	0.80 (0.17)	0.83 (0.20)	0.65
CRP (mg/dL)	0.08 (0.08)	0.14 (0.23)	0.69	0.08 (0.08)	0.04 (0.03)	0.16	0.09 (0.10)	0.17 (0.26)	0.39

Italicized values indicate the significant difference.

*∗*p < 0.05, Mann-Whitney *U* test, and Fisher exact test.

Parameter values are shown as the mean (standard deviation) or numbers.

BMI: body mass index; PBF, percent body fat.

**Table 5:** AUC, cutoff value, and sensitivity and specificity of the serum cystatin C level for prediction of the presence of osteoporosis.

	AUC	SE	p	95% CI	Cutoff value	Sensitivity, Specificity, %
Osteoporosis	0.683	0.087	0.042	0.512–0.855	0.840	50.0, 86.7

AUC: area under curve, SE: standard error, and CI: confidence interval.

## Data Availability

No data were used to support this study.
